# Mucinous Carcinoma in a Male: First Documented Case in Nicaragua

**DOI:** 10.7759/cureus.67674

**Published:** 2024-08-24

**Authors:** Gilberto A Altamirano, Christopher K Romero, Catherine S Moreno Cabrera, Lorenzo E Aragón Conrado

**Affiliations:** 1 Medical Education, Hospital Militar Escuela "Dr. Alejandro Dávila Bolaños", Managua, NIC

**Keywords:** multi-disciplinary teams, modified radical mastectomy, retro-areolar tumors, mucinous carcinoma, male breast cancer

## Abstract

Breast malignancy in men is an exceedingly rare condition, representing a small fraction of all diagnosed breast cancer cases. The most common histological subtype is invasive ductal carcinoma, while the mucinous type is extremely rare. This pathology has a high mortality rate due to its poor prognosis and diagnosis in advanced stages, often initially overlooked with limited screening. Surprisingly, more men have died from breast cancer than from testicular cancer. This report details a case of invasive mucinous carcinoma in a 75-year-old male who presented with a five-week history of chronic non-productive cough and signs of pleural effusion. A breast magnetic resonance imaging (MRI) revealed a retroareolar breast tumor, and a second-look ultrasound confirmed the presence of a BI-RADS 4C solid nodule. Histopathological and immunohistochemical results were confirmed by ultrasound-guided tru-cut biopsy, identifying invasive mucinous carcinoma and luminal B (HER2+) subtype. Staging studies were negative for metastasis, and a modified radical mastectomy was performed, yielding favorable intraoperative findings. The incidental diagnosis in this patient highlights the necessity of comprehensive imaging in atypical presentations. Despite its rarity, awareness and early detection of mucinous carcinoma are essential for optimizing patient outcomes. This case also underscores the disparity in breast cancer outcomes between low gross domestic product (GDP) and high-GDP countries, emphasizing the need for improved access to diagnostic and therapeutic resources. Enhanced clinical awareness and early detection are crucial for improving outcomes in patients with rare histological subtypes, particularly in underserved regions.

## Introduction

Male breast cancer is a rare entity, constituting 0.2% of all male cancers and less than 1% of all new breast cancer cases [1]. Its incidence increases with age [2], with nearly 75% of cases occurring in men aged 60 years or older [3].

Pathologies that contribute to a hormonal profile with excess estrogen increase the risk of developing this neoplasm. Risk factors include excess estrogen, obesity, alcohol consumption, liver disease, and Klinefelter syndrome [4]. BRCA2 mutations are highly significant, with an incidence rate of up to 10% among male carriers [5,6].

Approximately 85% of male breast cancers are histologically invasive ductal carcinoma, while mucinous, medullary, papillary, tubular, and cribriform subtypes comprise less than 10% [1]. Treatment typically involves mastectomy and axillary lymph node dissection, with systemic therapy guidelines being similar for both sexes, yielding comparable response rates.

## Case presentation

A 75-year-old male with a medical history notable for insulin-dependent diabetes mellitus, chronic arterial hypertension, both controlled KDIGO G5 chronic kidney disease on hemodialysis, and a body mass index of 36.72, furthermore has a family history of maternal left renal adenocarcinoma.

**Figure 1:**
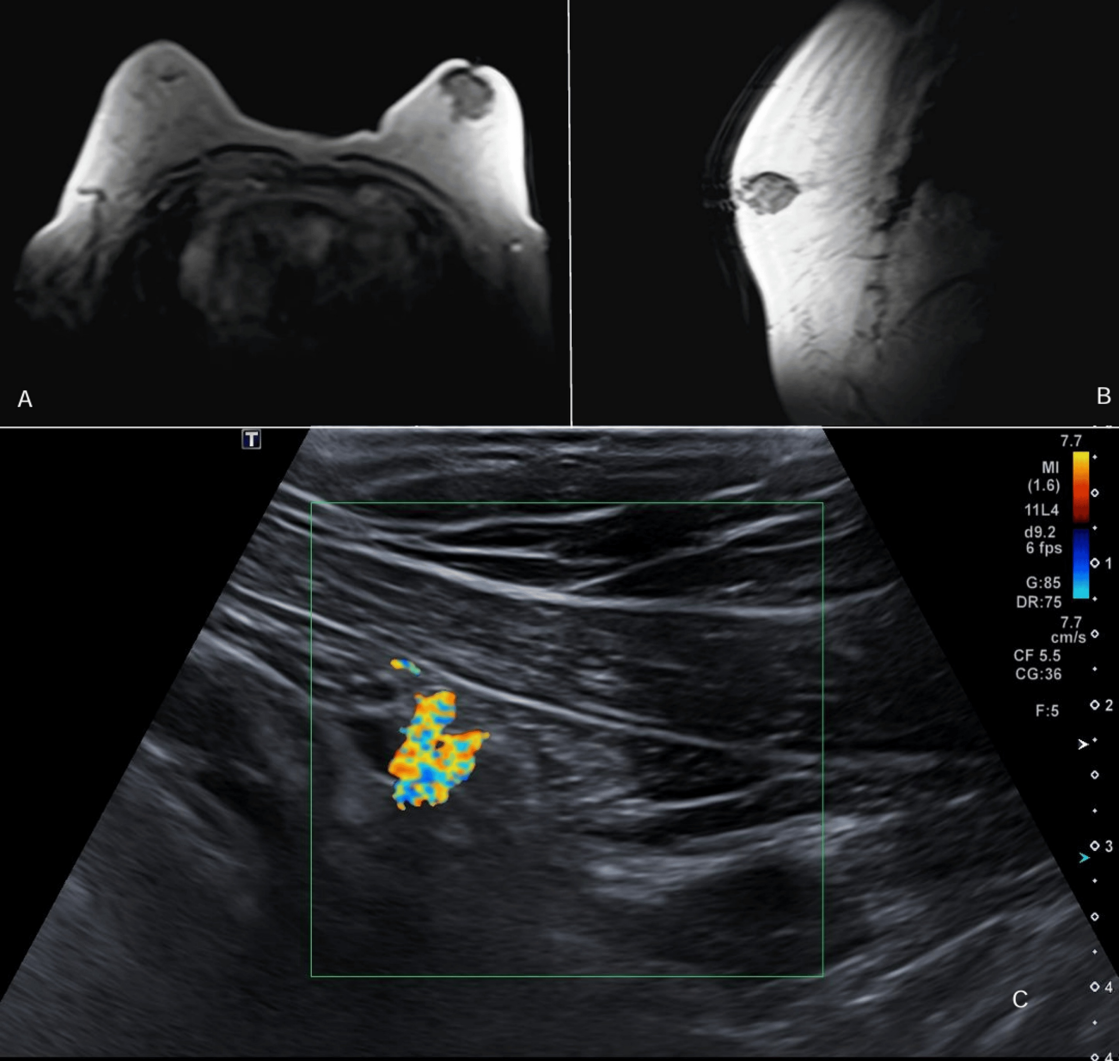
(A,B) Magnetic resonance imaging and (C) ultrasound of the breast (A,B) A hypodense lesion in the left breast suggestive of a nodule with irregular characteristics, angulated margins, and solid components. (C) The nodule measures approximately 40.4 × 26.3 × 43 mm, showing penetrating vascularity on color Doppler and minimal acoustic enhancement.

The patient presented with a five-week history characterized by chronic, non-productive cough and dyspnea without any specific time of day for symptom exacerbation. Physical examination revealed dullness to percussion and an absence of vesicular murmur in the left fifth and sixth intercostal spaces, consistent with fluid interposition syndrome, and a 3 cm diameter retroareolar mass in the left breast, non-mobile and petrous. A computed tomography (CT) scan incidentally detected a tumor with neoplastic features in the retroareolar region of the left breast. However, no significant findings are observed in the lung fields, mediastinum, or major vessels. Given the tumor’s characteristics, a more detailed imaging study was warranted. Breast magnetic resonance imaging (MRI) revealed irregular tissue with well-defined borders in the left retroareolar region (Figures 1A, 1B). Subsequent ultrasound reassessment confirmed the vascularity of the left breast nodule (Figure 1C), categorized as BI-RADS 4C.

**Figure 2:**
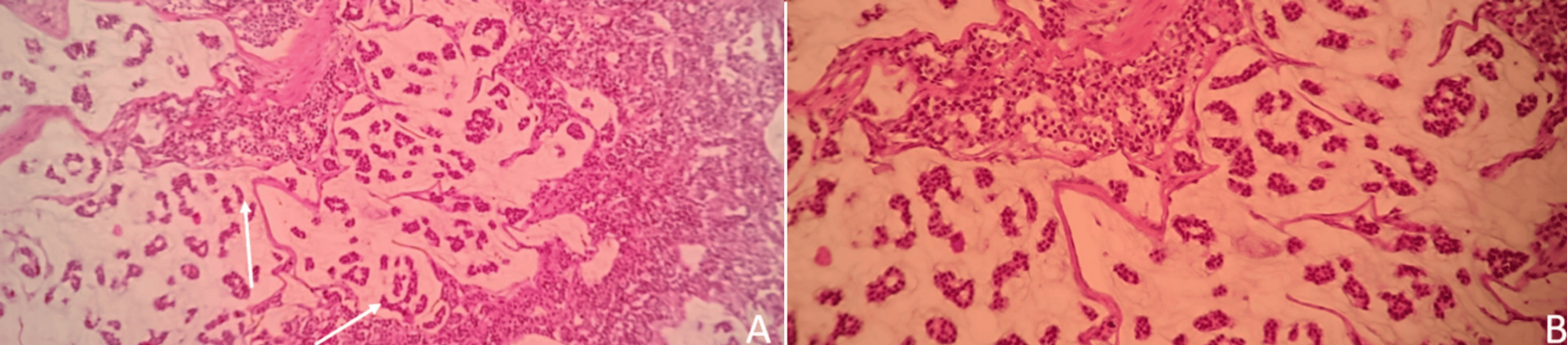
Histopathological findings of the left breast biopsy (A) Low power (4×) view (white arrows) showing clusters of tumor cells immersed in a mucinous matrix. (B) Medium power (10×) view showing neoplastic cells with round to oval nuclei and eosinophilic cytoplasm forming pseudoglandular structures within a mucinous background.

Ultimately, the diagnosis was confirmed through an ultrasound-guided tru-cut biopsy. Histopathological examination revealed invasive mucinous carcinoma with over 50% mucin content within the tumor (Figures 2A, 2B). Immunohistochemical analysis indicated positivity for estrogen receptor, progesterone receptor, HER2/neu, and a Ki67 proliferation index of 60%, categorizing this breast cancer molecularly as luminal B HER2-positive subtype.

Once the diagnosis was confirmed, the oncology department proceeded with the standard laboratory analysis panel (Table 1). Significant findings included hypercalcemia, mild hypochloremia, elevated creatinine, hypochromic anemia, increased BUN, and slight thrombocytopenia; these abnormalities are attributable to the patient’s underlying chronic conditions.

**Table 1:** Laboratory tests performed with results and reference values MCV, mean corpuscular volume; MCH, mean corpuscular hemoglobin; MCHC, mean corpuscular hemoglobin concentration; AST, aspartate aminotransferase; ALT, alanine aminotransferase; PTT, partial thromboplastin time; PT, prothrombin time

Laboratory test	Results	Reference range
Hematocrit	34.1%	39-50%
Hemoglobin	10.5 g/dL	13-17 g/dL
Red blood cell (count)	4.47 10e6/µL	4-6.3 10e6/µL
MCV	82.8 fL	72-96 fL
MCH	25.9 pg	27-32 pg
MCHC	30.8 g/dL	32-37 g/dL
Lymphocytes (count)	1.59 10e3/µL	20-45 10e3/µL
Lymphocytes %	17.2%	25-35%
Neutrophils (count)	6.77 10e3/µL	5.5-6.5 10e3/µL
Neutrophils %	73.4%	55-65%
Eosinophils (count)	0.13 10e3/µL	3-5 10e3/µL
Platelets	138 10e3/µL	150-500 10e3/µL
RDW-CV	15.8%	11-16%
RDW-SD	47.4%	37.2-54%
Immature granulocytes	0%	0-2%
Inorganic phosphorus	5.54 mg/dL	2.7-6.7 mg/dL
Glucose	82.1 mg/dL	82-115 mg/dL
Magnesium	2.19 mg/dL	1.63-2.41 mg/dL
Blood urea nitrogen (BUN)	42.88 mg/dL	7.8-21.4 mg/dL
Total urea	91.81 mg/dL	16.7-45.9 mg/dL
Potassium	4.6 mmol/L	3.5-5.1 mmol/L
Sodium	138.1 mmol/L	136-145 mmol/L
Calcium	11.46 mg/dL	8.4-9.6 mg/dL
AST	11.92 U/L	0-39 U/L
ALT	5.6 U/L	7-63 U/L
Total bilirubin	0.51 mg/dL	0-1 mg/dL
PTT	11.5 sec	9.6-12 sec
TPT	33.9 sec	22.7-32.5 sec
Creatinine	8.87 mg/dL	0.7-1.2 mg/dL

Staging studies revealed no evidence of metastasis, resulting in a clinical staging of T2N0MX. Radiographs of the upper limbs, spine, hips, and femur showed no abnormalities. A 2 cm diameter blastic lesion was identified in the frontal region of a skull radiograph, which, given the absence of nodal involvement and chronic kidney disease, was considered secondary to hyperparathyroidism. 

**Figure 3:**
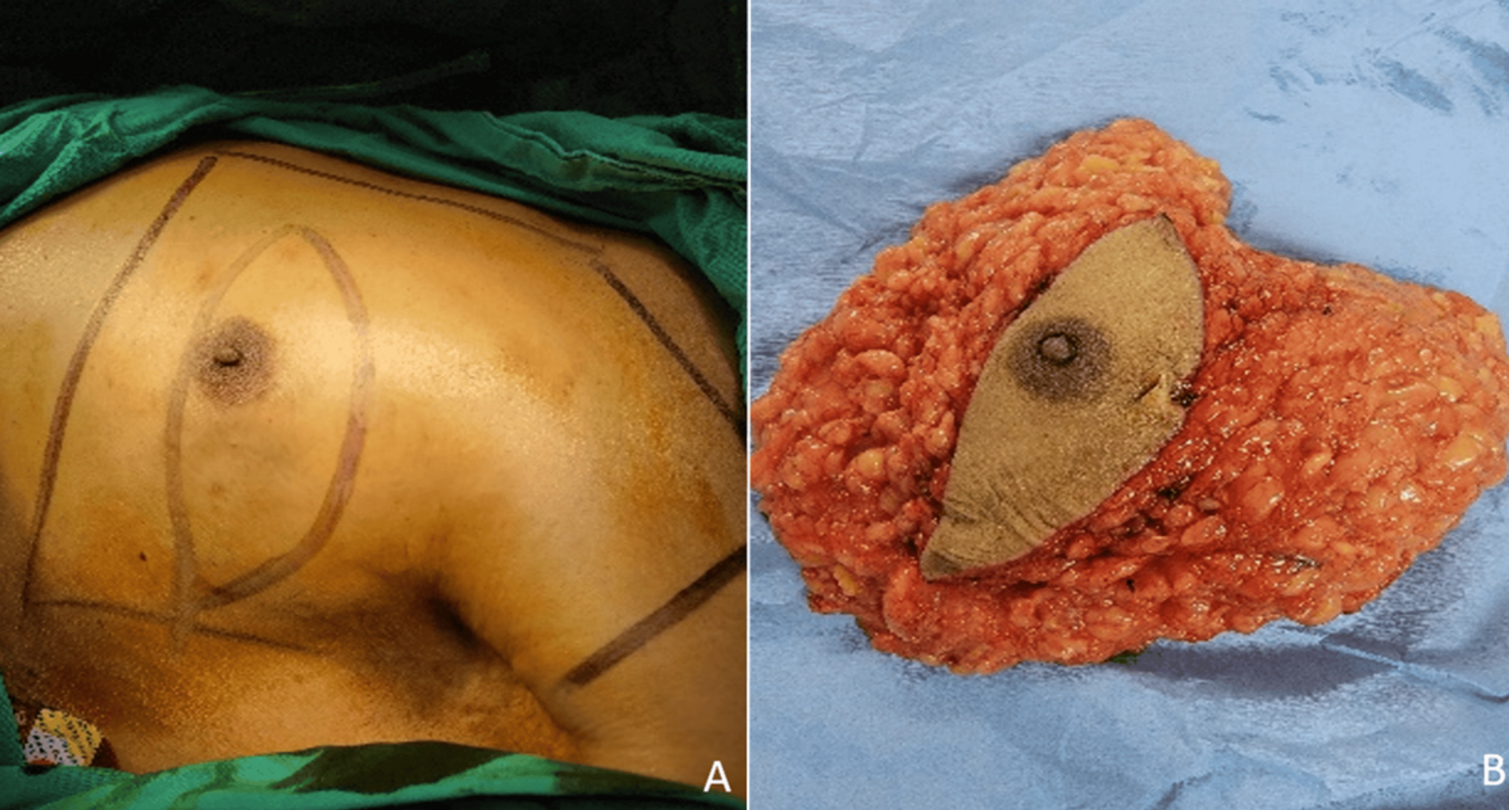
(A) Preoperative findings of a 3 cm retroareolar tumor and (B) excision of the left mammary gland

The patient underwent a modified radical left mastectomy (Figure 3A), with an excision of the mammary gland sent for pathology (Figure 3B). Intraoperative findings included a normal-sized left lymph node chain with no infiltration into the pectoral muscle, subcutaneous tissue, or skin. Subsequently, the patient was evaluated by gynecologic oncology for management due to his chronic kidney disease. Chemotherapy will not be administered, and the administration of tamoxifen was being considered; it has been decided to start solely with HER2 inhibitors to assess the patient’s tolerance. Subsequently, the possibility of introducing tamoxifen will be reassessed. The treatment consists of pertuzumab 420 mg and trastuzumab every 21 days for a total of 18 doses. Tumor markers such as cancer antigen 15-3 (CA 15-3) and carcinoembryonic antigen (CAE) will be used for monitoring. The five-year survival rate due to the cancer alone is 60%; however, the presence of associated comorbidities may worsen the prognosis.

## Discussion

Embryologically, mammary glands originate from mesoderm and ectoderm, becoming epithelium and stroma, respectively. Males do not experience further breast development during puberty due to high testosterone levels but can undergo hormonal changes throughout life, expressing androgen, progesterone, and estrogen receptors [7,8].

Male breast cancer is a rare entity, with an estimated 2,800 new cases in the United States in 2024, representing only 0.5-1% of total diagnosed cases [9]. The American Cancer Society estimates that at least 530 American men will die from this condition. Black men continue to have higher incidence and worse outcomes [10].

We present a case of mucinous carcinoma diagnosed in Nicaragua, a country with a poor prognosis. Latin American countries with lower gross domestic product (GDP) (Honduras, Nicaragua, and Haiti) show one death per 2.5 cases, compared to the United States or Canada, where there is one death per 6.5 cases. This disparity can be attributed to the lack of access to quality healthcare, diagnostic tools, and top-tier therapy for much of our population [11].

This case offers an opportunity to study a rare histological subtype of breast carcinoma. Histologically, 80% of new cases are ductal carcinoma, with the remainder being lobular. Ductal carcinoma is divided into non-specific and specialized types, including medullary, metaplastic, apocrine, cribriform, tubular, pleomorphic, neuroendocrine, and the rare mucinous type [12].

Also known as mucinous or colloid carcinoma, this malignant neoplasm features epithelial cells with abundant extracellular mucin, hyperpigmented nuclei, and indistinguishable nucleoli. There are two types: the more frequent pure type, which must contain at least 90% mucin, subdivided into hypercellular (with tubular, cribriform, papillary, micropapillary, and cord growth patterns) and hypocellular (with a unique nest pattern), and the mixed type, which contains less mucin. The partial type includes 30-50% mucin, and the mixed proper type contains 50-90% mucin [13].

Macroscopically, its characteristics resemble a benign lesion, presenting as a palpable retroareolar mass with a texture like adipose tissue and defined margins [14].

The etiology is considered multifactorial, with significant risk factors in this case being age, excessive estrogen exposure, and genetics. 

Incidence varies by age group. A study of 10,596 patients diagnosed with mucinous carcinoma over 12 years found that the predominant age group was ≥60 years, representing 68% of the sample [15]. Komenaka et al. reviewed 65 cases of pure mucinous carcinoma, finding an average age of 67 years [16]. These reviews support that the most affected age group is ≥60 years, to which the patient in this case belongs. Only 1% of cases occur in individuals under 35 years [17].

The biological relationship between aging and cancer-associated cellular changes may explain this marked predisposition in older ages. The accumulation of senescent cells involves the gradual loss of homeostasis mechanisms, as many of these cells exhibit “senescence-associated secretory phenotypes,” capable of producing cytokines like IL-1, IL-2, IL-6, and IL-8, as well as proteases and growth factors, leading to a pro-inflammatory environment that affects metabolic and immune function. Additionally, older age correlates with more telomere shortening, more years of UV radiation exposure, and endogenous reactive oxygen species, contributing to a pro-carcinogenic environment [18]. 

Estrogens induce ductal mammary proliferation; hence, increased estrogen or decreased androgens can stimulate abnormal mammary growth. Conditions increasing estrogen include Klinefelter syndrome, obesity, endocrine disorders, liver dysfunction, and certain drugs. Pathologies decreasing androgens, such as cryptorchidism, orchiectomy, and hormone replacement, also lead to increased estrogens [19]. 

Genetics plays a significant role in male breast cancer development, with 20% of patients having a first-degree relative with breast cancer. A third of these cases involve BRCA1/2 mutations. Approximately 5-10% of men with BRCA2 develop breast cancer, compared to 1-2% with BRCA1. Interestingly, the phenotype of BRCA1/2-related breast cancers in men seems more aggressive than in women. The AR gene plays a role in the transformation of male breast epithelial cells into neoplasms, expressed in up to 74% of male breast cancer cases.

Imaging of male breast cancer typically includes mammography and breast ultrasound. Male breast carcinoma most commonly presents as a high-density irregular mass on mammography. Secondary findings include calcifications, skin thickening, nipple retraction, and axillary adenopathy. Ultrasonographic features of invasive ductal carcinoma in men include discrete hypoechoic masses [20].

## Conclusions

Breast carcinoma in male patients, a rare condition predominantly affecting older men, poses distinctive challenges in both diagnosis and management, particularly magnified by healthcare access discrepancies evident in lower-income countries. This case study of mucinous carcinoma highlights the imperative of recognizing histological subtypes beyond the more prevalent ductal and lobular variants. The unique features of mucinous carcinoma, characterized by a mucin-rich cellular makeup and a macroscopic appearance akin to benign lesions, underscore the intricacies involved in early detection and precise diagnosis. Mucinous carcinoma is more prevalent among older individuals, indicating a significant age-related predisposition. Aging contributes to the accumulation of senescent cells and associated inflammatory responses, impacting cancer development. Understanding these age and molecular associations is critical for advancing targeted prevention and therapeutic strategies for affected populations.

Key imaging techniques such as breast MRI and ultrasound play a pivotal role in detecting these cancers early, providing essential insights into tumor characteristics that inform personalized treatment strategies. The incidental detection of the tumor in our case highlights the importance of comprehensive diagnostic assessments in male patients with unusual symptoms, guaranteeing prompt intervention and favorable results. Addressing disparities in male breast cancer outcomes between high- and low-GDP countries, such as Nicaragua, is crucial for improving global survival rates. The favorable intraoperative and postoperative outcomes observed in this patient following a modified radical mastectomy demonstrate the effectiveness of surgical intervention in managing male breast cancer.

## References

[1] Cardoso F, Bartlett JM, Slaets L (2018). Characterization of male breast cancer: results of the EORTC 10085/TBCRC/BIG/NABCG International Male Breast Cancer Program. Ann Oncol.

[2] Giordano SH (2018). Breast cancer in men. N Engl J Med.

[3] Özkurt E, Tükenmez M, Yılmaz R (2018). Favorable long-term outcome in male breast cancer. Eur J Breast Health.

[4] Bhardwaj PV, Gupta S, Elyash A, Teplinsky E (2024). Male breast cancer: a review on diagnosis, treatment, and survivorship. Curr Oncol Rep.

[5] Frank TS, Deffenbaugh AM, Reid JE (2002). Clinical characteristics of individuals with germline mutations in BRCA1 and BRCA2: analysis of 10,000 individuals. J Clin Oncol.

[6] Deb S, Jene N, Fox SB (2012). Genotypic and phenotypic analysis of familial male breast cancer shows under representation of the HER2 and basal subtypes in BRCA-associated carcinomas. BMC Cancer.

[7] Fox S, Speirs V, Shaaban AM (2022). Male breast cancer: an update. Virchows Arch.

[8] Ahmed U, Wagner S, Jordan S (2022). Mucinous carcinoma in a male patient: diagnosis and management of breast cancer in male patients. Radiol Case Rep.

[9] Siegel RL, Giaquinto AN, Jemal A (2024). Cancer statistics, 2024. CA Cancer J Clin.

[10] (2024). American Cancer Society. Cancer Facts & Figures. https://www.cancer.org/content/dam/cancer-org/research/cancer-facts-and-statistics/annual-cancer-facts-and-figures/2024/2024-cancer-facts-and-figures-acs.pdf.

[11] (2024). Organización Mundial De La Salud. Cáncer de mama en las Américas [Internet]. Organización Panamericana De La Salud. https://www.paho.org/sites/default/files/Cancer-mama-Americas-factsheet-ES%20%281%29.pdf.

[12] Malhotra GK, Zhao X, Band H, Band V (2010). Histological, molecular and functional subtypes of breast cancers. Cancer Biol Ther.

[13] Lu K, Wang X, Zhang W (2020). Clinicopathological and genomic features of breast mucinous carcinoma. Breast.

[14] Marrazzo E, Frusone F, Milana F (2020). Mucinous breast cancer: a narrative review of the literature and a retrospective tertiary single-centre analysis. Breast.

[15] Zhou X, Zheng Z, Li Y, Zhao W, Lin Y, Zhang J, Sun Q (2021). The clinical features and prognosis of patients with mucinous breast carcinoma compared with those with infiltrating ductal carcinoma: a population-based study. BMC Cancer.

[16] Komenaka IK, El-Tamer MB, Troxel A (2004). Pure mucinous carcinoma of the breast. Am J Surg.

[17] Garcia-Hernandez I, Lopez-Garcia CA, Cardona-Huerta S (2018). Pure mucinous breast carcinoma in a 25-year-old female, a case report. Int J Surg Case Rep.

[18] Berben L, Floris G, Wildiers H, Hatse S (2021). Cancer and aging: two tightly interconnected biological processes. Cancers (Basel).

[19] Łukasiewicz S, Czeczelewski M, Forma A, Baj J, Sitarz R, Stanisławek A (2021). Breast cancer-epidemiology, risk factors, classification, prognostic markers, and current treatment strategies-an updated review. Cancers (Basel).

[20] El Amrani S, Hiba Z, Hosni A, Omor Y, Latib R (2023). Synchronous bilateral breast cancer: a first case with lobular and mucinous carcinoma. Radiol Case Rep.

